# Selective Plasmonic Responses of Chiral Metamirrors

**DOI:** 10.3390/nano14211705

**Published:** 2024-10-24

**Authors:** Chang-Ruei Li, Yu-Wei Liao, Rashid G. Bikbaev, Jhen-Hong Yang, Lu-Hsing Chen, Dmitrii N. Maksimov, Pavel S. Pankin, Ivan V. Timofeev, Kuo-Ping Chen

**Affiliations:** 1 Institute of Lighting and Energy Photonics, College of Photonics, National Yang Ming Chiao Tung University, 301 Gaofa 3rd Road, Tainan 71150, Taiwan; e1253400@gmail.com; 2 Institute of Photonic System, College of Photonics, National Yang Ming Chiao Tung University, 301 Gaofa 3rd Road, Tainan 71150, Taiwan; yuwei880404@gmail.com (Y.-W.L.); s87069@hotmail.com (J.-H.Y.); 3 Kirensky Institute of Physics, Federal Research Center KSC SB RAS, Krasnoyarsk 660036, Russia; batblr_90@mail.ru (R.G.B.); mdn@tnp.krasn.ru (D.N.M.); p.s.pankin@mail.ru (P.S.P.); ivan-v-timofeev@ya.ru (I.V.T.); 4 Institute of Engineering Physics and Radioelectronics, Siberian Federal University, Krasnoyarsk 660041, Russia; 5 Institute of Photonics Technologies, National Tsing Hua University, Hsinchu 30013, Taiwan; wilson20000426@yahoo.com.tw; 6 Institute of Imaging and Biomedical Photonics, College of Photonics, National Yang Ming Chiao Tung University, 301 Gaofa 3rd Road, Tainan 71150, Taiwan

**Keywords:** metamirrors, circular dichroism, chirality, visible wavelengths

## Abstract

The properties of circularly polarized light has recently been used to selectively reflect chiral metasurfaces. Here we report the more complete basic functionalities of reflectors and absorbers that display various optical phenomena under circularly polarized light at normal incidence as before. For the chiral metamirrors we designed, the circular dichroism in about 0.4 reflection is experimentally observed in visible wavelengths. The experimental results also show high reflectance for right-handed circular polarization with preserved handedness and strongly absorbed left-handed circular polarization at chiroptical resonant wavelengths. By combining a nanobrick and wire grating for our design, we find and offer a new structure to demonstrate the superposition concept of the phase in the same plane that is helpful in effectively designing chiral metamirrors, and could advance development of their ultracompact optical components.

## 1. Introduction

The term chirality is identified as the property of an object that lacks any mirror symmetry plane [1,2,3,4,5,6,7] and is a fundamental characteristic of natural molecules and artificial metamaterials which have different optical properties for right-handed circular polarization (RCP) or left-handed circular polarization (LCP). A famous example in nature, the Chrysina gloriosa, a jeweled scarab beetle, can selectively reflect left-handed circularly polarized light in reflection, because of the particular textures of its exoskeleton [8]. Moreover, this chiroptical response is utilized by Chrysina gloriosa to perceive and communicate with its companions [9]. However, the signal of chiroptical response is universally weaker in natural as compared to artificial metamaterial. Therefore, in recent years the chiral metasurface [10,11,12,13] has been widely studied to achieve a strong chiroptical response in applications such as circular polarizers [14,15,16,17,18], hot-electron collection devices [19,20,21,22,23], optical encryption [24,25], and biosensors for analyzing circular dichroism spectroscopy [26,27,28,29,30].

**Figure 1:**
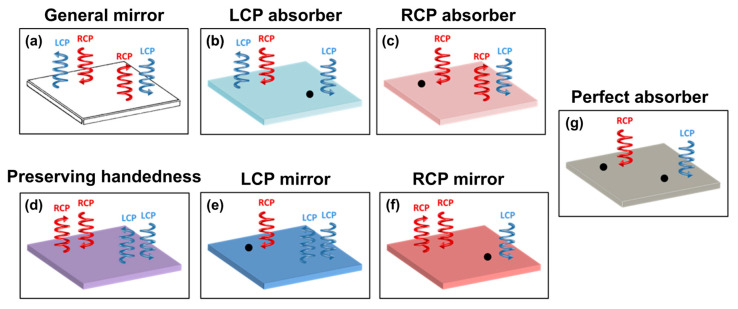
Schematic of all reflectors under circularly polarized light at normal incidence. (**a**) A general mirror reverses the handedness of circular polarized light in reflection. (**b**) An LCP absorber and (**c**) an RCP absorber can reflect circularly polarized light of one handedness with handedness variation, while absorbing the other handedness, as shown as the black circle. (**d**) An anisotropy mirror is designed as a half-wave plate with a phase difference of π, to preserve handedness without handedness variation. (**e**) An LCP mirror and (**f**) an RCP mirror can reflect circular polarized light of one handedness and preserve handedness without handedness variation, while absorbing the other handedness. (**g**) An isotropic mirror absorbs both LCP and RCP light and does not reflect light as a perfect absorber.

In this paper, we demonstrate all basic functionalities for a series of reflectors and absorbers consisting of a metasurface on the top of a conventional mirror under circularly polarized light at normal incidence (as shown in Figure 1) that is more complete than before [31,32]. A conventional isotropic mirror can reverse the handedness of circularly polarized when light is reflected off its surface, because it can reverse the propagation direction of the reflected electromagnetic wave of the electric field (seen in Figure 1a), as has previously been discussed in detail [33,34]. The necessary condition for selective reflection without preserving handedness for both LCP absorbers and RCP absorbers under circular polarized light is only to break mirror symmetry to the perpendicular light propagation direction of the pattern plane, illustrated in Figure 1b,c. In addition, Figure 1b,c are opposite phenomena and mirror images of each other. Figure 1d shows an anisotropy mirror designed as a half-wave plate with a phase difference of π [35,36,37,38] and having the property of preserving handedness without handedness variation. The LCP and RCP mirrors are designed to not only selectively reflect one circular polarized light while preserving handedness, but also totally absorb the other, as shown in Figure 1e,f [30]. In addition, a linear polarization perfect absorber composed of a metasurface and a thick backplane of a general mirror separated by a thin lossy dielectric spacer, has been reported [39,40]. It is important to note that an isotropic linear polarization absorber can absorb both linear polarizations [41,42,43], and is the circular polarization perfect absorber to absorb both RCP and LCP light, as shown Figure 1g. The mirrors shown in Figure 1 allow for control of the polarization and intensity of the reflected light, which is essential when designing various devices. This study aims to develop a metamirror with the maximum value of circular dichroism. The metamirror analysis revealed that circular dichroism can only be obtained by using LCP or RCP mirrors (see Figure 1e,f). For this reason, in this work we present a simple approach that utilizes the superposition concept of the phase to design chiral metamirrors from a single structure layer, by combining the nanobrick and the wire grating in the same plane. Furthermore, this single patterned layer can make a good contribution to lowering the complexity of the fabricated device and its cost. The analysis of both simulation and experiments with reflectance coefficients spectra demonstrates the chiral metamirrors we have designed can selectively reflect the RCP light while preserving the handedness and absorbing the LCP light. In addition, the simulated results display good agreement with the experimental results showing reflectance spectra and spectral shape.

## 2. Materials and Methods

**Figure 2:**
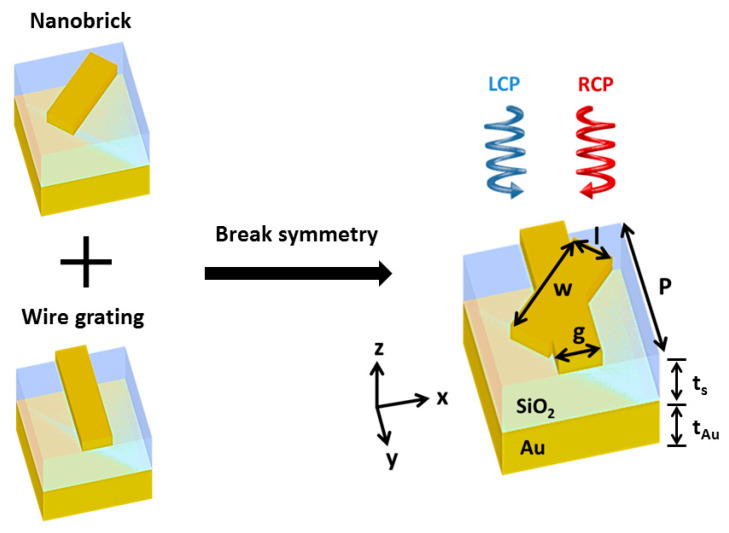
Schematic configuration of the chiral metamirrors consisting of the nanobrick and the wire grating in the same plane for breaking mirror symmetry and the *n*-fold (*n* > 2) rotational symmetry. A unit cell of the metasurface consists of a gold structure which is separated from a thick gold reflector by a thin SiO_2_ spacer. Geometrical parameters: P = 250 nm, g = l = 70 nm, w = 190 nm, t_s_ = 130 nm, t_Au_ = 200 nm.

In order to design the circular dichroism metamirrors, conditions including breaking mirror symmetry and the *n*-fold (*n* > 2) rotational symmetry [8] must be satisfied. Therefore, we combined the nanobrick and the wire grating in the same plane. Figure 2 shows a three-dimensional illustration of the gold nanobrick–wire grating complex structure. The schematic configuration is designed as a 60 nm gold structure on top of a 130 nm silicon dioxide spacer, underneath which is a layer of thick gold reflector and a glass substrate. Due to the optically thick gold reflector in this design, transmission can be neglected; the chiroptical response can be tremendously enhanced by providing a resonant cavity. The width of both the nanobrick and the wire grating was g = l = 70 nm, and the length of the nanobrick was w = 190 nm. The unit cell was replicated in a two-dimensional square lattice along the x and y axes with period Px = Py = P = 250 nm. The nanobrick was rotated by 45 degrees with respect to the z-axis to break the mirror symmetry and the *n*-fold (*n* > 2) rotational symmetry. It is worth noting that we demonstrate that the gold nanobrick-wire grating complex structure of the chiral metamirrors planar photonic structures with a single patterned layer not only produces a chiral optical response, but also lowers the complexity of a fabricated device and the cost.

## 3. Results

### 3.1. Simulated Results

**Figure 3:**
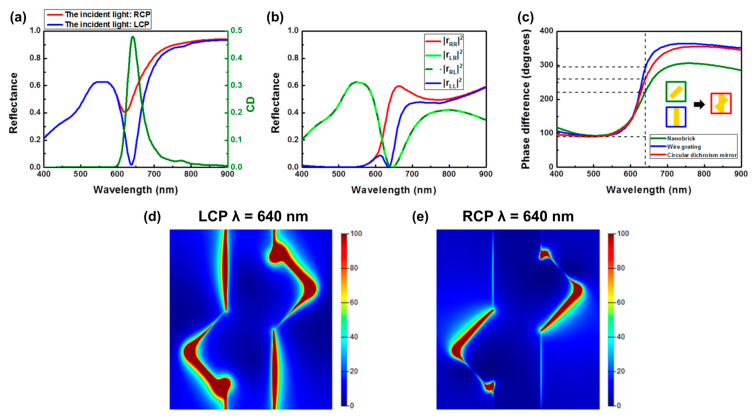
Simulated results of the chiral metamirrors. (**a**) Total reflectance spectra and (**b**) reflectance coefficients of the co-polarization and cross-polarization components of the chiral metamirrors under RCP and LCP incident light. (**c**) Phase difference of reflected light for the three types of structure under circularly polarized light. Electric field distributions for the chiral metamirrors at the maximum chiroptical response wavelength of 640 nm under (**d**) LCP and (**e**) RCP incident light.

Simulated results of the reflectance spectra under RCP and LCP incident light based on the three-dimensional Finite-Difference Time-Domain method are illustrated in Figure 3a,b. To better describe the observed chiroptical response, the corresponding circular dichroism is illustrated in Figure 3a. The circular dichroism spectrum was characterized by different absorption between RCP and LCP light (CD = A_LCP_ – A_RCP_). The total reflectance spectra indicated clearly different reflection under RCP and LCP incident light, and the circular dichroism was ~0.48 at a wavelength of 640 nm. In contrast to the phenomenon of the RCP light, which was reflected, the LCP was absorbed by our chiral metamirrors. Moreover, the corresponding reflecting behavior of the reflectance coefficients of the co-polarization and cross-polarization components of the chiral metamirrors under RCP and LCP incident light is depicted in Figure 3b. The reflectance coefficient |r_RR_|^2^ (|r_LR_|^2^) is defined as the RCP (LCP) light reflected off the surface of the chiral metamirrors under RCP incident light, while |r_RL_|^2^ (|r_LR_|^2^) indicates the cross polarization of circularly polarized light. As expected, |r_RL_|^2^ and |r_LR_|^2^ were exactly the same because of the symmetry of the unit cell [44]. In addition, Figure 3b demonstrates our chiral metamirrors not only selectively reflected the RCP light, but also preserved the handedness at the chiroptical resonance wavelength of 640 nm, while all LCP components were completely absorbed. In order to preserve handedness, there is a need to design the anisotropy structure with a phase difference of π as a half-wave plate. For our designed dichroism metamirrors, the superposition concept of the phase is illustrated in Figure 3c. First, we modified two different lengths of brick to create two kinds of phase difference. Then, we combined these two structures to superpose the phase to create the phase difference of π, to preserve the handedness that is one of the necessary conditions for designing chiral metamirrors. Consequently, we have successfully demonstrated this simple way to design chiral metamirrors which produce the chiroptical response while preserving handedness by combining the nanobrick and the wire grating in the same plane. The chiral selective reflectance spectra demonstrated the distinct resonance modes under the RCP and LCP incident light. To better describe the fundamental physical mechanism of the chiral resonance at a wavelength of 640 nm while the value of circular dichroism is maximal, the cuts in the electric field distribution through the middle of the gold metasurface were as shown in Figure 3d,e. In the near field distribution, the electric field localizes to a different position, which means the resonance mode is different, resulting in different reflectance spectra. Furthermore, it is evident that the stronger field restriction on both sides of long axis of the nanobrick corresponded to the lower reflectance under LCP light.

In principle, the circular polarizations can be decomposed into two linear polarizations with orthogonal direction in a 90° phase shift. The anisotropy of our designed structure can produce the phase difference and the transformation of the linear polarization state into its orthogonal one. This phase difference will lead to destructive interference and constructive interference for linear polarizations under LCP and RCP light, respectively. The ideal conditions of the phase and the amplitude for maximizing circular dichroism (maximizing LCP absorption, minimizing RCP absorption) in the planar chiral metamaterial are as follows [19]:

*ϕ_xx_* + *90*° = *ϕ_xy_* = *ϕ_yx_* = *ϕ_yy_* + 270° (1)

|*r_xx_*| = |*r_yy_*| = |*r_xy_*| = |*r_yx_*| (2)

**Figure 4:**
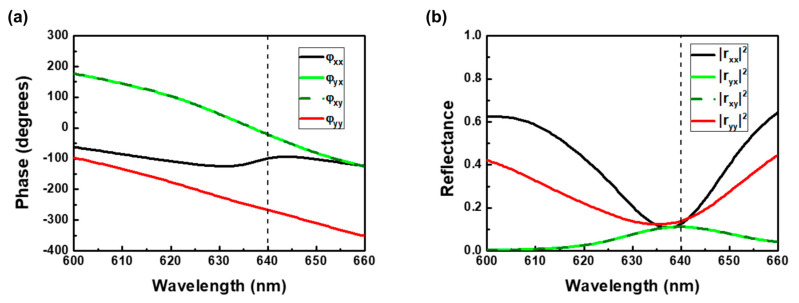
Simulated results of the chiral metamirrors. (**a**) Phase and (**b**) amplitude of reflectance spectra under linearly polarized light.

As mentioned above, our chiral metamirrors have also satisfied these two conditions in achieving the maximizing circular dichroism at the resonant wavelength of 640 nm as shown in Figure 4. As shown in Equation (2) and Figure 4b, the same amplitude will not only cancel out the same linear polarizations of the reflected light for destructive interference, but also enhance the intensity of the other polarization for constructive interference. A more detailed description of the process for achieving the required phase difference and producing a half-wave plate metasurface has been presented in our previous work [38].

### 3.2. Experimental Results

**Figure 5:**
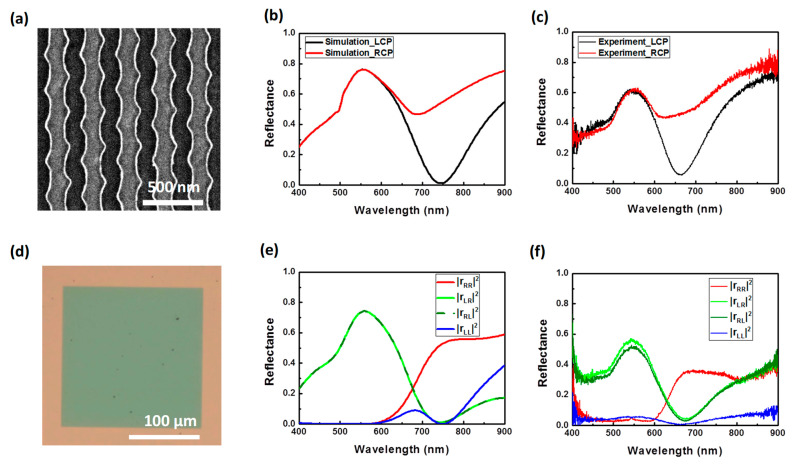
Experimentally measured results of optical reflectance spectra of the chiral metamirrors with a thin adhesion layer of titanium. (**a**) Scanning electron microscopy image of the fabricated sample of the chiral metamirrors. The scale bar is 500 nm. (**b**) Simulated and (**c**) experimental results of the total reflectance spectra under LCP (black) and RCP (red) incident light. (**d**) Optical microscopy images of the metamirrors show good uniformity in e-beam lithography. The scale bar is 100 μm. (**e**) Simulated and (**f**) experimental results of reflectance coefficients of the co-polarization and cross-polarization components of the chiral metamirrors under RCP and LCP incident light.

To experimentally realize the chiral metamirrors, we demonstrate a series of experiments in the visible wavelengths. E-beam lithography was used to fabricate the metasurface. First, the 200 nm gold film and 130 nm amorphous SiO_2_ were deposited via sputter on the glass substrate. The PMMA A4 photoresist with a thickness equal to 200 nm was spin coated on the substrate at 1500 rpm for 15 s and 8000 rpm for 30 s, respectively. Then, it was prebaked at 180°C for 90 s. The lower spin speed was for flatness, and higher spin speed for controlling thickness. The E-beam lithography system (ELS−7500EX, ELIONIX, Wellesley, MA, USA) was used to define the pattern, and the photoresist was developed using developers (MIBK:IPA = 1: 3) for 30 s. Then, 60 nm gold film was deposited using the electron gun evaporation system (VT1-10CE, ULVAC, Munich, Germany) with a 0.5 Å/s deposition rate in 5 × 10^−6^ Torr. The sample was immersed in acetone liquid to lift off residual photoresist and unnecessary gold film. During the gold deposition process, we encountered an issue with its adhesion to the SiO_2_ surface. To address this, we deposited a thin 5 nm titanium film on the SiO_2_ prior to applying the gold. The scanning electron microscope image of the fabricated structure is shown in Figure 5a. Figure 5c shows the measured total reflectance spectra at wavelengths between 400 nm and 900 nm under RCP and LCP incident light. A comparison of the experimental spectra with the theoretical ones (see Figure 3a) reveals that the Q-factor of the resonance in the actual experiment is significantly lower than in the simulation. This significant decrease in Q-factor is due to the inclusion of a thin titanium layer, which was necessary to enhance the adhesion between gold and SiO_2_. The simulated reflection spectra for the structure with a thin titanium film under RCP and LCP incident light are shown in Figure 5b. As expected, a clearly chiral selective reflection appears, as shown in Figure 5b,c. The minimum reflectance for LCP waves reached ~5% at the chiroptical resonant wavelength in the measurement. However, by comparing the simulated and experimental results we can observe the reflectance spectra under circular polarized light underwent a blue shift because of the wider width of the wire grating of ~15 nm in the experiment. Nevertheless, the experimental results match well with the simulated results. Figure 5e,f plot the reflectance coefficients of the co-polarization and cross-polarization components of the chiral metamirrors under RCP and LCP incident light, fully providing information on the chiroptical response. We observed the RCP light can be reflected with preserved handedness on our chiral metamirrors at the resonant wavelength of 670 nm, and the LCP components were absorbed almost simultaneously, as shown in Figure 5f. Nonetheless, the reflectance coefficients spectra also underwent a slight blue shift because of the inaccuracy from the sample fabrication, as mentioned above, and show a slight imprecision due to the optical components at longer than 800 nm.

## 4. Discussion

In summary, we have presented more complete basic functionalities of reflectors and absorbers than previously demonstrated, and an approach to the superposition concept of the phase in the same plane that is helpful in designing chiral metamirrors. We have also demonstrated the design of our metamirrors offers significant magnitudes of chiroptical response in both simulated and experimental results in the visible wavelengths. The theoretical reflectance is nearly absorbed at the chiroptical resonant wavelength under LCP incident light, while a reflectance of ~5% is experimentally proven. We believe that the high resolution for chiral selection in reflection is an attractive feature for many applications in optical components such as circular polarizers and absorbed filters. In addition, applying the characteristic of preserving handedness to circular polarizers could avoid transforming one polarization state into the other. This transformation can complicate an optical system and influence the measurement of samples in ways we do not expect. It is important to note that our designed single-patterned layer is a useful contribution to decreasing the complexity of fabrication requirements such as the high cost of a multilayered patterned layer [8] and the accurate alignment, and can also achieve a highly efficient chiroptical response. Finally, the proposed structure, consisting of nanowires coupled with nanobricks, can be used in the design of beam steering devices [45,46]. As in previous studies, the nanowires do not connect, allowing them to be used as electrical contacts and, consequently, to change the diffraction grating period. The advantage of this proposed chiral metamirror in contrast to conventional nanostrips is that it allows for the creation of a diffraction grating. The proposed metamirror allows control of the diffraction angles, as well as the intensity of diffraction orders and the polarization.

## Data Availability

The data presented in this study are available on request from the corresponding author.
